# Marinoid J, a phenylglycoside from *Avicennia marina* fruit, ameliorates cognitive impairment in rat vascular dementia: a quantitative iTRAQ proteomic study

**DOI:** 10.1080/13880209.2020.1837187

**Published:** 2020-12-05

**Authors:** Xiang-xi Yi, Jia-yi Li, Zhen-zhou Tang, Shu Jiang, Yong-hong Liu, Jia-gang Deng, Cheng-hai Gao

**Affiliations:** a Institute of Marine Drugs, Guangxi University of Chinese Medicine, Guangxi, China; b Faculty of Pharmacy, Guangxi University of Chinese Medicine, Guangxi, China

**Keywords:** ACE, neuron apoptosis, proteomics

## Abstract

**Context:**

Fruit of *Avicennia marina* (Forsk.) Vierh. (Acanthaceae) is used as a Chinese herb. Studies have found that it contains marinoid J, a novel phenylethanoid glycoside (PG) compound, but its neuroprotective functions are largely unknown.

**Objective:**

This study evaluated the effects of marinoid J on vascular dementia (VD) and determined its potential mechanisms of action.

**Materials and methods:**

The VD model was established by the ligation of the bilateral common carotid artery in Sprague–Dawley rats, who received daily intragastrically administration of saline, marinoid J (125 or 500 mg/kg body weight/d), or oxiracetam (250 mg/kg body weight/d) for 14 days (20 rats in each group). The Morris water maze (MWM) was used to evaluate cognitive performance. The hippocampus was subjected to histological and proteomic analyses.

**Results:**

Marinoid J shortened the escape latency of VD rats (31.07 ± 3.74 s, *p* < 0.05). It also decreased malondialdehyde (MDA) (27.53%) and nitric oxide (NO) (20.41%) while increasing superoxide dismutase (SOD) (11.26%) and glutathione peroxidase (GSH-Px) (20.38%) content in hippocampus tissues. Proteomic analysis revealed 45 differentially expressed proteins (DEPs) in marinoid J-treated VD rats, which included angiotensin-converting enzyme (ACE), keratin 18 (KRT18), cluster of differentiation 34 (CD34), and synaptotagmin II (SYT2).

**Conclusions:**

Marinoid J played a role in protecting hippocampal neurons by regulating a set of proteins that influence oxidative stress and apoptosis, this effect may thereby alleviate the symptoms of VD rats. Thus, pharmacological manipulation of marinoid J may offer a novel opportunity for VD treatment.

## Introduction

Vascular dementia (VD) is a form of senile dementia characterized by loss of cognitive function. Structural changes in the brain caused by cerebrovascular disease and other risk factors are considered to cause VD. Studies have shown that reduced cerebral blood flow results in oxidative stress, causing an increase in the levels of reactive oxygen species (ROS). A considerable number of unsaturated fatty acids are damaged by ROS, which consequently deteriorates the neural structure and function associated with learning and memory, leading to cognitive dysfunction (Shi et al. 2012; Manzanero et al. 2013). VD is the second most common cause of dementia after Alzheimer disease (Roman 2003). The main clinical symptoms of VD are impaired learning and cognition. In severe cases, ‘brain failure syndrome’ may occur. As the population ages, the number of VD patients has increased annually, which has placed a heavy economic burden on families and society (Matthews et al. 2013)_._

Clinically, memantine and the acetylcholinesterase inhibitors, donepezil and galantamine, exhibit certain efficacies in VD but are unable to treat the entire series of complications. In addition, acetylcholinesterase inhibitors have side effects such as nausea, vomiting, and even death (Bowler 2003). Because of the complexity of the pathogenesis of VD, drugs used for VD treatment have limited efficacy and can only alleviate some symptoms. Therefore, it is important to further clarify the pathogenesis of VD and to discover and develop more effective drugs.

In China, the leaves, fruits, bark gum, and liquids of the mangrove plant, *Avicennia marina* (Forsk.) Vierh. (Acanthaceae), have been used in folk medicine for contraception and treatment of abscesses and diarrhoea (Shao et al. 2009). Gao et al. (2014) found that *Avicennia marina* contains alkaloids, aromatic lipids, phenylethanoid glycosides (PGs), and other compounds. The main components of PGs are caffeic acid, phenylethanin, and glycosyl groups. In recent years, with the increased attention on PGs, research on these compounds has rapidly progressed. Currently, approximately 200 types of PGs have been isolated from different plants, and novel PGs are continuously being discovered. Numerous studies have shown that PGs have a variety of biological activities, such as enhancing immunity and exerting antibacterial and antioxidative effects. In addition, some studies have shown that PGs can improve neurocognitive function. For example, glycosides from *Cistanche deserticola* Y.C. Ma (Orobanchaceae) have been shown to improve learning and memory in a rat model of VD and to play a critical role in protecting hippocampal neurons by decreasing tau phosphorylation and increasing the expression of collapsin response mediator protein-2 (Chen et al. 2015)_._

In our previous work (Gao et al. 2014), marinoid J, a novel PG compound, was isolated from *Avicennia marina* for the first time_,_ but its biological activity has remained largely unknown, especially in terms of its neuroprotective functions.

In the present study, we investigated the effects of marinoid J on learning and memory in a rat model of VD and also performed proteomic analysis of the hippocampus to gain insights into the neuroprotective effects of marinoid J. Our results demonstrate that marinoid J ameliorated cognitive impairments in VD rats.

## Materials and methods

### Materials

Fresh ripe *Avicennia marina* fruits were harvested in Beihai City by our research group during 2017. The specimen was identified by Hangqing Fan from Guangxi Mangrove Research Centre, Guangxi Academy of Sciences, China. A voucher specimen (2017-GXUCM-006) was deposited in the Institute of Marine Drugs, Guangxi University of Chinese Medicine, China. HPD750, DA-201, DM-130, and AB-8 macroporous adsorption resins were purchased from Zhengzhou Qinshi Technology Co., Ltd. (Zhengzhou, China); echinacoside was purchased from Chinese Pharmaceutical Organisms Inspection Institute (Hangzhou, China); carbon-18 reverse-phase silica gel (Chromatorex, SMB100-20/45) was purchased from Fuji Silysia Chemical Ltd. (Kasugai Aichi, Japan); trypsin was purchased from Promega Corporation (Madison, WI); Calbiochem protease inhibitor was purchased from Shanghai Yubo Biotechnology Co., Ltd. (Shanghai, China); and a BCA Kit was purchased from Biyuntian Biotechnology Co., Ltd. (Shanghai, China). The Thermo Labelling Kit, acetonitrile, and triethylammonium bicarbonate buffer were purchased from Thermo Fisher Scientific (Waltham, MA); oxiracetam capsules were purchased from Shiyao Group Ouyi Pharmaceutical Co., Ltd. (Hebei, China); and penicillin potassium for injections was purchased from Harbin Pharmaceutical Group Veterinary Pharmaceutical Factory (Harbin, China). SOD, GSH-Px, NO, and MDA Kits were purchased from Nanjing Jiancheng Bioengineering Research Institute (Nanjing, China). The Waters-2695 High-Performance Liquid Chromatography System, using the Sunfire C18 column (150 × 10 mm i.d., 10 μm) coupled to a Waters 2998 photodiode array detector (Waters, Milford, MA), was used in the present study.

### Animals

Adult male Sprague–Dawley rats, weighing 240 ± 10 g, were purchased from Guangxi Medical University (SCXK Gui 2014-0002; Guangxi, China). Animals were housed under 12 h light and dark conditions with food and water available *ad libitum*. The experiment began after seven days of adaptive feeding. This study was conducted in accordance with the recommendations of the Guide for the Care and Use of Laboratory Animals and was approved by the Ethics Committee for Animal Experimentation and Use Committee of Guangxi University of Chinese Medicine (Animal study ethic number: 201710014).

### Animal groupings and drug administration

Sprague–Dawley rats were randomly divided into five groups: sham (*n* = 20), VD (*n* = 20), VD + olracetam (*n* = 20), VD + marinoid J high-dose (*n* = 20), and VD + marinoid J low-dose (*n* = 20). The high-dose and low-dose groups were intragastrically administered marinoid J at 500 and 125 mg/kg/d, respectively, for dose selection, we refer to some work of others (Bag et al. 2013; Chen et al. 2015); the positive control group was intragastrically administered oxiracetam solution at 250 mg/kg/d (Xu et al. 2019). The sham and VD groups were intragastrically administered equal volumes of normal saline per day for a total of 14 d. Animals were given free access to food and water throughout the study.

### Preparation of the VD model VD

Rat VD model (Chen et al. 2015; Jia et al. 2015; Yang et al. 2018) was used in the study, briefly, rats were anaesthetised by intraperitoneal injection of 10% chloral hydrate (350 mg/kg) and placed on an alcohol-sterilized surgical plate. The bilateral common carotid arteries were separated from the vagus nerve and ligated with a 4-0 surgical suture. The wound was disinfected with iodophor after suturing. The sham group was treated the same as the other groups, but without artery occlusion. Penicillin (2000 U/d) was continuously injected for three days after surgery.

### Morris water maze test

To assess learning and memory, rats were trained and tested in the Morris water maze (MWM) (Yang et al. 2018). The platform was fixed in the northeast quadrant, and the east, west, south, and north were used as the starting positions; training was conducted four times a day for five consecutive days. Rats were placed facing the pool wall, and swimming started at each starting point until they found and climbed onto the platform, the total time of which was recorded as the escape latency. On the sixth day after the start of training, a spatial exploration experiment was performed. Briefly, the platform in the water was removed, the rats were placed into the water from the starting point and were allowed to swim freely for 120 s, and the quadrant dwell time in the target quadrant was recorded.

### Histology of the hippocampal CA1 area

After completion of all training and testing in the MWM, the rats were anaesthetised, their brains were harvested and placed in a 4% paraformaldehyde solution, and paraffin blocks were prepared by dehydration and waxing via conventional steps to prepare sections of brain tissue. The morphologies of neurons in the hippocampal CA1 region were observed and examined under a light microscope (Jia et al. 2015; Yang et al. 2018).

### Detection of the oxidative stress ability index

After completion of training and testing in the MWM, the rats were anaesthetised, their brains were quickly harvested, brain tissue was dissected, and pre-cooled physiological saline was added at 1:9 (w:v) to prepare a 10% homogenate. After centrifugation, the supernatant was removed for further analysis. SOD activity, GSH-Px activity, MDA content, and NO content were determined according to the instructions of each corresponding kit as previously reported (Li et al. 2017), and protein concentrations were determined by the BCA method.

### Proteomic analysis

For proteomic analysis, data detection and analysis were commissioned by Hangzhou Jingjie Biotechnology Co., Ltd (Hangzhou, China). The isobaric tag for relative and absolute quantification (iTRAQ) with high-resolution liquid chromatography-tandem mass spectrometry (LC-MS/MS) analyses was performed as previously reported (Yang et al. 2018). The main experimental steps were as follows. First, for protein extraction, at the end of the experiment, rats were anaesthetised, and their brains were quickly harvested and mixed with lysis buffer. The mixture was sonicated three times on ice via a high-intensity ultrasonic processor. The resulting suspension was centrifuged at 12,000 *g* for 10 min, the supernatant was collected and considered as total protein, and the total protein concentration was determined by the BCA method. Second, for protein alkylation and enzymatic digestion, the extracted protein (100 μg) in lysis buffer was adjusted to a final volume of 100 μL. Then, 50 mM of DTT solution was added to each sample, which was then placed in a sterile incubator for 30 min at 56 °C. Next, IAM solution was alkylated for 15 min at 37 °C in the dark. The urea concentration of each sample was diluted to less than 2 M after alkylation. Trypsin was added at a ratio of 1:50 and digested at 37 °C for 16 h. Then, trypsin was added again at a ratio of 1:100, digestion was continued for another 4 h, and the supernatant was collected for analysis of digested peptides. Third, for identification of proteins via nano LC-MS/MS, peptides were labelled with a TMT Labelling Kit and subjected to nano LC-MS/MS. Peptides were loaded onto an EASY-n LC 1000 Ultra High Liquid Phase System maintaining a flow rate of 800 nL/min. Then, the eluted peptide was loaded in a 2.0-kV NSI ion source for ionisation, with a capillary temperature set to 200 °C. Peptide ions were detected using a high-resolution Orbitrap Fusion Tribrid Mass Spectrometer. First-order MS was obtained in the MS mode at 350–1800 *m/z* with a resolution of 70,000. The secondary MS scan started at 100 *m/z* with a resolution of 35,000. The 20 most intense precursor ions were selected for collision-induced fragmentation with a normalised collision energy of 30% in a linear ion trap. Dynamic exclusion was used within 30 s to prevent repeated scanning of the parent ions.

### Database search

The resulting MS/MS data were processed using Maxquant (v.1.5.2.8). MS/MS with a reverse database connection was searched in the UniProt database (http://www.uniprot.org/). Trypsin/P was designated as the cleaving enzyme. The mass tolerance of the precursor ion was set to 20 ppm in the first search and 5 ppm in the main search, and the mass tolerance of the fragment ion was set to 0.02 Da. The carbamoyl group on cysteine was designated as a fixed modification, and the oxidation on methionine was designated as a variable modification. The false discovery rate was adjusted to <1% and the lowest score of the peptide was set to >40.

### Statistical analysis

All statistical analyses were conducted using SPSS 20.0 analysis software (SPSS Inc., Chicago, IL). Group differences in the escape latency in the Morris water maze task were analysed by using two-way ANOVA with repeated measures, the factors being group and time. Otherwise, data were analysed by one-way ANOVA followed by the Tukey’s *post hoc* test. Significance was set at *p* < 0.05 for all tests.

## Results

### Isolation and purification of marinoid J from *Avicennia marina*

**Figure 1.**
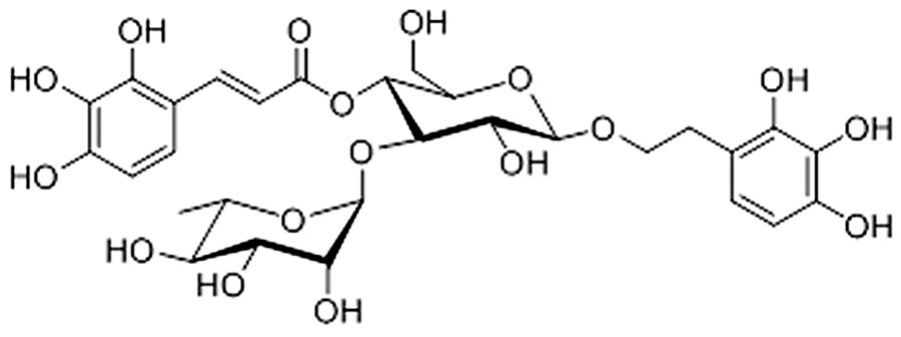
Molecular structure of marinoid J.

The fruits of *Avicennia marina* were smashed and immersed in 95% ethanol, the extract was concentrated and dried, and the macroporous adsorption resin and carbon-18 reversed-phase silica gel were used to isolate and purify PGs. When eluted with 40% ethanol at a flow rate of 6 mL/min, PGs with a purity of 76.68% were obtained. PGs were subjected to Sephadex LH-20 column chromatography with CHCl_3_–MeOH (1:1) and were then separated via HPLC using mixtures of MeOH–H_2_O (45:55) to isolate marinoid J (Figure 1).

### Effects of marinoid J on learning and memory in VD rats

**Figure 2.**
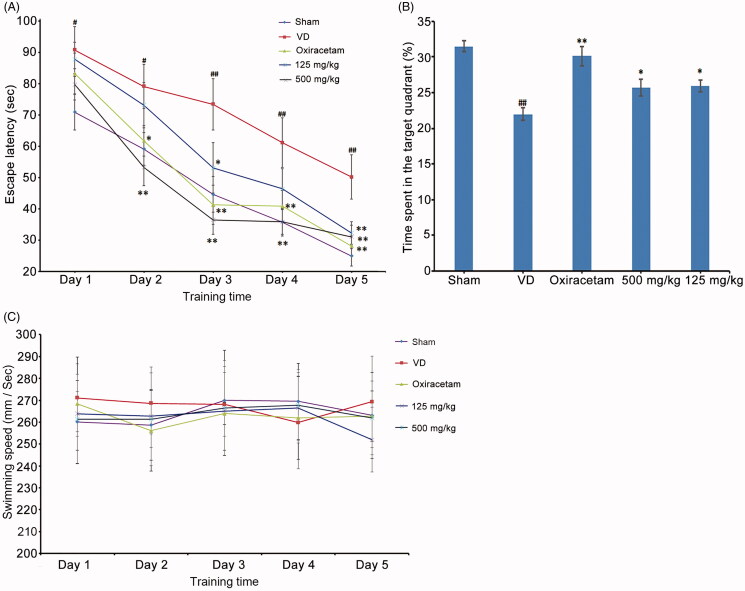
Marinoid J from (A) marina ameliorates the cognitive and behavioural impairment of VD rats. (A) Escape latency time from the MWM test in each group. (B) Percentage of time spent in the quadrant for each group of rats. (C) Swimming speed of rats during five days in hidden platform trial. All data are presented as the mean ± SD (*n* = 10). #*p* < 0.05, ##*p* < 0.01 vs. sham group. *p < 0.05, **p < 0.01 vs. VD group.

To evaluate the effects of marinoid J on learning and memory in VD rats, we trained and tested rats in the MWM. It was found that the escape latency decreased significantly during training (*F* = 33.43, *p* < 0.01) and there was a significant difference among the groups (*F* = 5.38, *p* < 0.01). Compared with that of the VD group, the escape latencies of marinoid J groups (i.e., low dose and high dose) were significantly shortened. As can be seen from Figure 2(A), on training day 2, 500 mg/kg marinoid J treatment significantly decreased the escape latency of VD rats (*n* = 10, *p* < 0.01). On the third day, the escape latency was significantly decreased in both marinoid J treated groups (*n* = 10, *p* < 0.01), on the fifth day after the start of MWM training, the escape latency of the high-dose marinoid J group was shortened to 31.07 ± 3.74 s. In the probe trial, the VD group exhibited a shorter time in the target quadrant compared to that of the sham group (22.00 ± 3.12% vs. 31.508 ± 2.70%, *p* < 0.01). After treatment with marinoid J, the time spent in the target quadrant was also increased (Figure 2(B)). However, the swimming speed was unaffected by surgery or marinoid J, indicating that these rats had equivalent motor activity (Figure 2(C)). These results demonstrate that marinoid J ameliorated cognitive and behavioural deficits of VD rats.

### Effects of marinoid J on hippocampal CA1 neurons in VD rats

**Figure 3.**
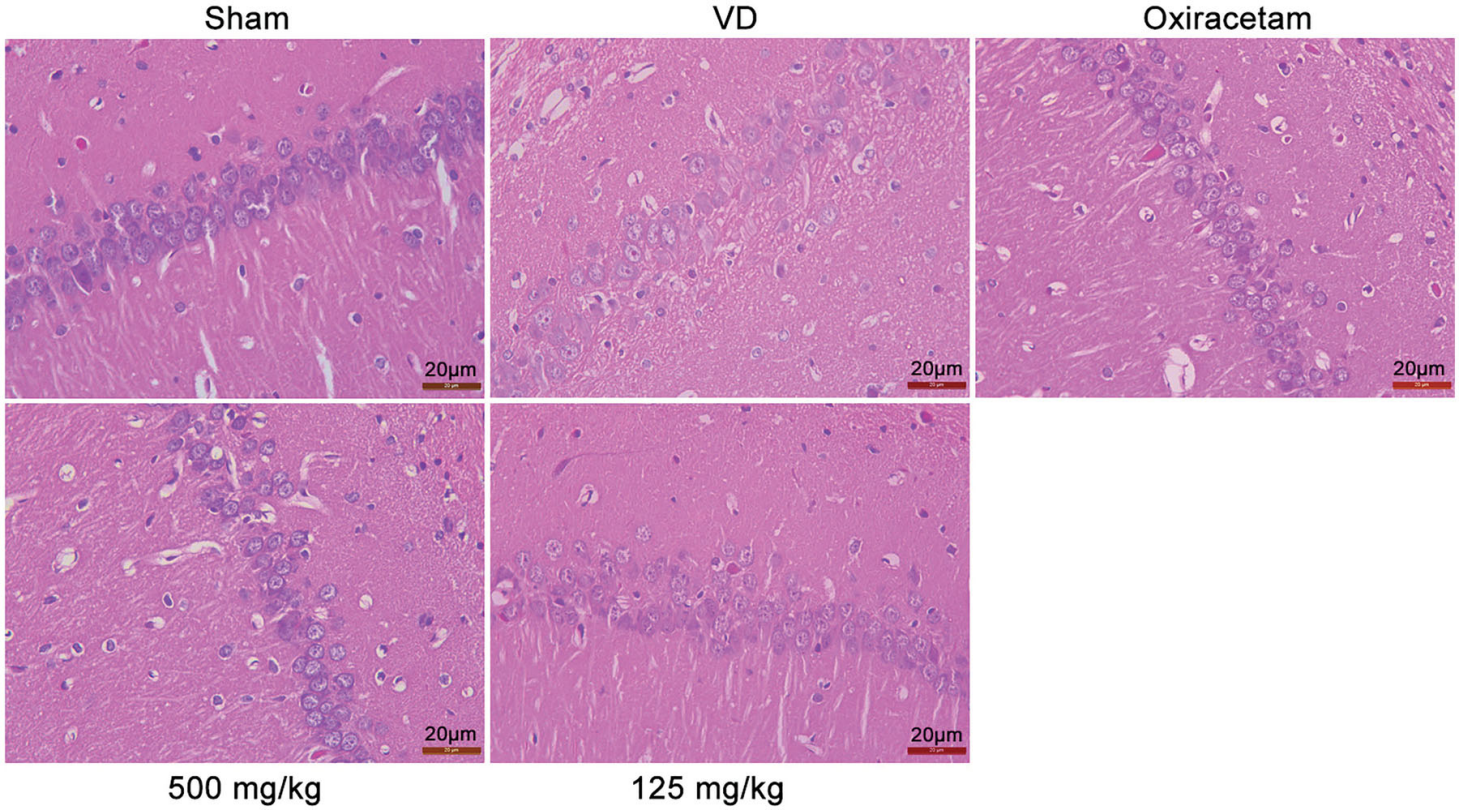
Morphological analysis of neuronal cells in the hippocampal CA1 region of the rat brain in each group (HE staining, magnification × 400).

To evaluate the neural-specific effects of marinoid J in rats, we performed histopathological examination of the hippocampal CA1 region in the rat brain; the results are shown in Figure 3. In the absence of sufficient blood supply, the structure of the hippocampal CA1 region of VD rats was damaged, the nucleus was condensed, the nuclear border was unclear, and the number of necrotic cells was increased. After treatment with marinoid J, damage to the hippocampal CA1 area in the marinoid J group was significantly reduced. These results show that marinoid J reduced neuronal damage caused by ischaemia.

### Effects of marinoid J on oxidative stress in the brains of VD rats

**Figure 4.**
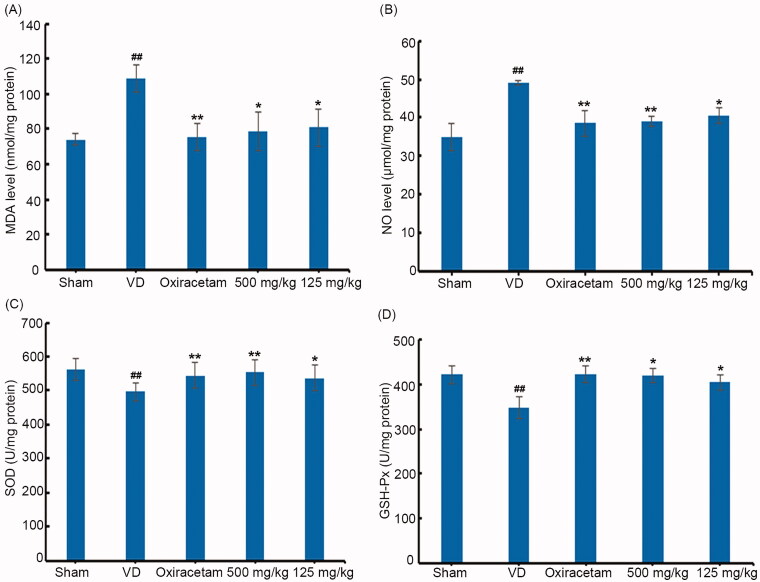
Marinoid J reduce the oxidative stress in the brain of VD rats. Marinoid J decrease the level of MDA (A) and NO (B) in the brain of VD rats. Marinoid J restore the activities of both SOD (C) and GSH-Px (D) in the brain of VD rats. All data are presented as the mean ± SD (*n* = 8). #*p* < 0.05, ##*p* < 0.01 vs. sham group. *p < 0.05, **p < 0.01 vs. VD group.

We examined the levels of MDA and NO, as well as the activities of GSH-Px and SOD, in the brains of VD rats. Compared to parameters in sham rats, MDA and NO levels were increased, and GSH-Px and SOD enzymatic activities were decreased in VD rats. After treatment with high-dose marinoid J (500 mg/kg), MDA levels were decreased by 27.53% (*n* = 8, *p* < 0.05), NO levels was decreased by 20.41% (*n* = 8, *p* < 0.01), while SOD activity was increased by 11.26% (*n* = 8, *p* < 0.01), SOD activity was increased by 20.38% (*n* = 8, *p* < 0.05) (Figure 4(A–D)). These results indicate that marinoid J could reduce oxidative stress in the brains of VD rats.

### Analysis of differentially expressed proteins (DEPs) in the hippocampi of VD rats

Through proteomic analysis, a total of 5902 proteins were identified, of which 5183 had quantitative information and the total number of peptides was 42,755. After the MS data were searched, the quantitative proteins were screened based on analyzable proteins, and a protein-change ratio ≥1.2-fold between groups was set, and proteins with *p* < 0.05 were identified as differentially expressed proteins (DEPs). Comparison of the VD group and the Sham group identified 34 DEPs, among which 22 were up-regulated and 12 were down-regulated. Comparison of with VD group and MJ group identified 45 DEPs, among which 25 were up-regulated and 20 were down-regulated. In the marinoid J group, the upregulated DEPs included ACE and KRT18, whereas the downregulated DEPs included CD34 and SYT2 (Figure S2).

### Gene ontology enrichment of DEPs in the hippocampus

**Figure 5.**
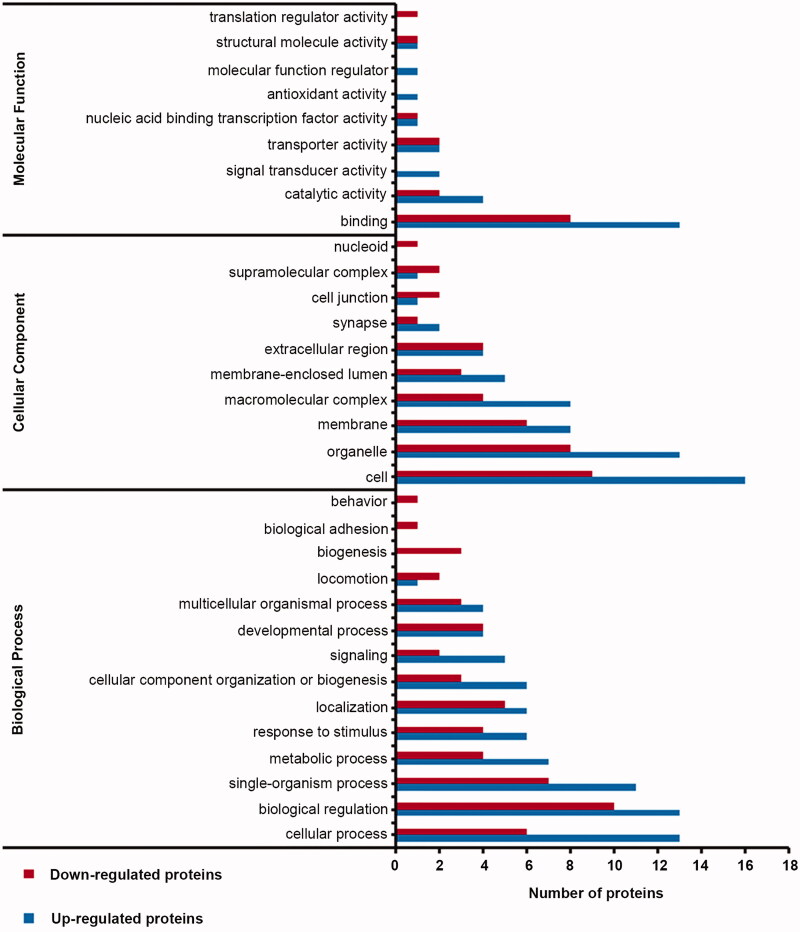
Distribution of gene ontology secondary markers for DEPs in the VD group.

**Figure 6.**
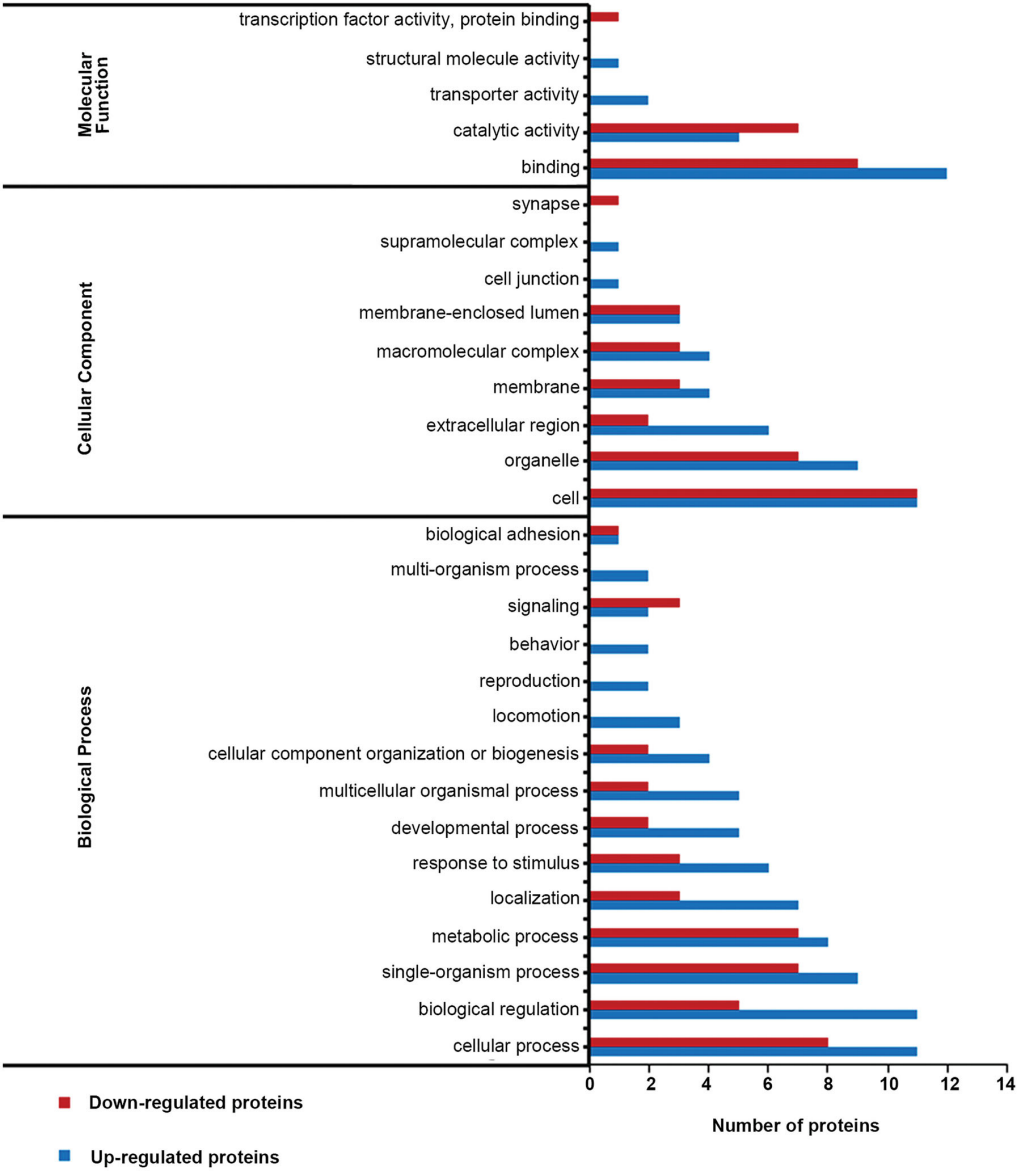
Distribution of gene ontology secondary markers for DEPs in the marinoid J group.

To investigate the biological effects of DEPs identified by proteomic analysis, we performed Gene Ontology (GO) analysis. In the VD group, 34 DEPs were involved in binding, catalytic activity, and other processes (Figure 5). In the marinoid J group, a total of 37 DEPs were matched to molecular functions, and the participating molecular functions included binding, catalytic activity, transport, protein activity, structural molecular activity, and transcription-factor activity (Figure 6). The cellular components included synapses, supramolecular complexes, and cellular junctions. Finally, the included biological processes were biological adhesion, signalling and behaviour.

### Kyoto encyclopedia of genes and genomes (KEGG) pathway analysis of DEPs in the hippocampus

Kyoto Encyclopaedia of Genes and Genomes (KEGG) pathway analysis showed that a total of 34 DEPs in the VD group were mapped to 47 pathways. The main enriched pathways were the transient receptor potential pathway (TRP) (ko04750) (Figure S3) and the advanced glycation end product (AGE)-receptor for AGE signalling pathway (ko04933) (Figure S4). A total of 118 signalling pathways were retrieved from 45 DEPs in the marinoid J group. The main enrichment pathways were the TRP pathway (ko04750) (Figure S5) and pathways associated with Alzheimer’s disease (ko05010) (Figure S6), Parkinson’s disease (ko05012) (Figure S7), and Huntington’s disease (ko05016) (Figure S8).

## Discussion

PGs are a type of glycosidic compound consisting of caffeic acid, phenylethanin, and glycosyl, and they are widely found in the roots, bark, and leaves of some dicotyledons. PGs have been reported to exert antioxidative, antitumor, and immune-regulatory activities. Studies have shown that PGs are efficacious in the treatment of neurological diseases. For example, previous research (Wang et al. 2009) found that acetonide of *Verbascum sinuatum* L. (Scrophulariaceae) reduced the production ROS and inhibited cytochrome C release and caspase-3 clearance, thereby inhibiting the cytotoxicity in SH-SY5Y cells caused by amyloid (Aβ)25-35. Currently, the effects of PGs from *Avicennia marina* fruits on VD have not been reported.

In the present study, marinoid J was obtained by separating and purifying it from the fruits of *Avicennia marina.* Based on behavioural and histopathological studies, we found that marinoid J significantly ameliorated learning and memory deficits and reduced neuronal damage in VD rats, which is consistent with findings from previous reports. In addition, marinoid J also significantly reduced MDA and NO levels in the brains of VD rats, and increased SOD and GSH-Px activities.

The hippocampal CA1 region, which is sensitive to hypoxia-ischaemia in the brain, is the functional zone most closely related to episodic/declarative learning and memory (Burda et al. 2005). Many studies have shown that neuronal apoptosis in the hippocampal CA1 region of VD rats is severe, which may represent the pathological basis of VD. We found that marinoid J significantly decreased neuronal apoptosis in VD rats, thereby alleviating the symptoms of VD.

The pathogenesis of VD is a complicated process, and conventional research methods have not been able to fully elucidate its many components. Proteomics can be used for large-scale studies of proteins at the structural and functional levels to obtain a global view of cellular metabolism, disease development, and other processes. Therefore, the application of proteomic technology is of great value for the prevention, diagnosis, pathogenesis, and drug discovery of various diseases (Dayon et al. 2011; Ning et al. 2011; Datta et al. 2013). To date, there have been a large number of reports on the application of proteomic technology for exploring the pathogenesis of cerebrovascular diseases. A previous study (Dhodda et al. 2004) used two-dimensional combined with matrix-assisted laser desorption/ionisation-time of flight analysis to determine that the expression levels of HSP70, HAP27, HAP90, platelet-activating factor receptor, β-actin, and guanylate cyclase were increased in the ischaemic rat brain. Another recent study (Van der Ende et al. 2019) also used unbiased MS to reveal 20 DEPs between symptomatic mutation carriers and noncarriers, as well as nine DEPs between symptomatic and presymptomatic carriers. These studies have helped to further elucidate the underlying mechanisms of marinoid J in ameliorating changes in overall protein levels in VD. Furthermore, the identification of various DEPs is beneficial for the discovery and selection of novel drug targets for VD.

To investigate the mechanisms by which marinoid J exerts neuroprotective effects in VD, we employed proteomic analysis to identify DEPs in the hippocampi of VD rats. We found that several proteins related to amino acid transport, neural tube formation, vascular development, energy metabolism, and signalling pathways showed differential expression in the hippocampus of VD rats treated with marinoid J compared to those in sham rats treated with saline. These DEPs included ACE, KRT18, CD34, and SYT2.

ACE is an important member of the renin-angiotensin system that converts angiotensin I (Ang I) into Ang II. ACE has vasoactive and aldosterone-activated effects and plays an important role in maintaining fluid balance and blood pressure stability. In the brain, Ang II binds to two receptors: AT1 and AT2. AT2 receptors primarily mediate the recall of learning acquisition and passive avoidance behaviour, predominating in learning and memory, and protecting cognitive function (Braszko 2005; Steckelings et al. 2005). In addition, Ang II exerts protective effects against vasoconstriction, inflammation, and neuronal apoptosis by binding to AT2 receptors (Xu et al. 2011; Jiang et al. 2013). In the present study, we found that marinoid J treatment promoted the expression of ACE. These results indicate that marinoid J may exert protective effects by increasing the expression of ACE to inhibit neuronal apoptosis.

KRT18 encodes the type-I intermediate filament chain, keratin 18. KRT18 is perhaps the most commonly found member of the intermediate filament gene family. It is expressed in single-layer epithelial tissues throughout the body. Aberrant expression or mutations in this gene have been linked to disease, such as kidney disease (Lebherz-Eichinger et al. 2013). Currently, little is known about the role of KRT18 in VD. However, according to a recent study (Alpua and Kisa 2019), caspase-cleaved keratin 18 (CCCK-18) can be used as a prognostic biomarker for Alzheimer’s disease. Further comprehensive studies should be performed to clarify the use of CCCK-18 levels as a biomarker for other neuronal diseases.

CD34 is a highly glycosylated transmembrane glycoprotein that is mainly expressed on the surface of human haematopoietic stem cells. It is also the molecular basis of important pathophysiological processes involved in immune responses and inflammation. During inflammation, CD34 works with E-selectin and other proteins, through side-chain binding to leukocyte surface receptors, to mediate aggregation of leukocytes, initiation of inflammatory responses, as well as to cooperate with chemokines to enhance inflammatory responses (Epstein et al. 2006). Studies have shown that CD34 is abnormally expressed during inflammation, especially in chronic inflammatory diseases (Blanchet et al. 2007, 2010). In our present study, the upregulation of CD34 in VD rats and relative CD34 downregulation after PG treatment suggests that PGs may exert anti-inflammatory effects *in vivo*.

Additionally, CD34-positive cells (CD34^+^) have been recognised to play a role in the maintenance of microvasculature. Decreased levels of bone-marrow-derived circulating CD34^+^ cells correlate with vascular dysfunction (Hill et al. 2003). Patients with decreased levels of CD34^+^ cells display significant worsening in neurologic function; hence, levels of circulating CD34^+^ cells have prognostic value for neural function (Taguchi et al. 2009). To some extent, a decreased number of CD34^+^ cells can be mirrored by down-regulated CD34 proteins. In our present study, CD34 was highly expressed in VD rats, which may seem to contradict a neuroprotective function of CD34. After carotid artery ligation, blood flow to the brain decreases. As a self-regulating mechanism, VD rats may respond to reduced blood flow by promoting more capillary angiogenesis. As such, the VD-induced increase in CD34 expression that we observed may indicate enhanced angiogenesis following VD.

Signal transmission at the neuromuscular junction is mediated via the release of acetylcholine from synaptic vesicles. This process is rendered calcium-sensitive by members of the Synaptotagmin family, which also has roles in vesicle priming and in reducing spontaneous neurotransmitter release (Lee et al. 2008). SYT2 is the major isoform expressed at the neuromuscular junction, and previous studies have shown that *Syt2* knockout mice show markedly reduced calcium-evoked neurotransmitter release (Pang et al. 2006). A previous study (Whittaker et al. 2015) found that *Syt2* mutations cause a novel and potentially treatable complex presynaptic congenital myasthenic syndrome characterized by motor neuropathy inducing lower-limb wasting and foot deformities. A recent study (Bereczki et al. 2018) used in-depth proteomics to compare 32 post-mortem human brains in the prefrontal cortex of prospective AD patients, PD patients with dementia, dementia patients with Lewy bodies, and older adults without dementia. They found a significant loss of SYT2, which implicates that it could be a synaptic marker of cognitive decline in neurodegenerative diseases. Our present study showed that abnormal expression of SYT2 may be related to the occurrence of VD, and PGs may decrease the expression of SYT2 in VD rats, thereby alleviating the symptoms of VD rats.

In our present study, a comparative analysis of KEGG pathways showed that the number of DEPs in the VD group was greater than that in the PG group, and these DEPs were found to be involved in many complex signalling pathways. In the VD group, TRP pathway members, protein kinase C (PKC), and PKCδ were upregulated. In contrast, in the marinoid J group, PKCδ, PKC, and adenosyl cyclase were downregulated. TRP pathway members are widely expressed, can be activated through many different mechanisms, and play important roles in various cellular functions. For example, TRP pathway members, including proteins such as transient receptor potential cation channel subfamily V member 2, PKC, phosphatidylinositol 3-kinase, and insulin growth factor 1, are critical for the pathogenesis of several human diseases such as type-I diabetes, Alzheimer’s disease, cardiomyopathy, and myasthenia gravis syndrome (Nilius and Voets 2005; Nilius 2007; Akopian 2016).

PKC plays an important role in regulating many different biological processes. PKCδ is one of the isoenzymes of PKC and is a substrate for the pro-apoptotic protein, caspase-3 (Ghayur et al. 1996). Studies have shown that PKCδ is associated with apoptotic signals, and overexpression of PKCδ promotes apoptosis (Datta et al. 1997). In VD rats in the present study, the TRP signalling pathway was activated and PKCδ expression was increased, which correlated with increased apoptosis of hippocampal neurons. After treatment with marinoid J, PKCδ levels were downregulated, which correlated with inhibition of apoptosis. Collectively, our findings demonstrate that marinoid J promoted the survival of hippocampal neurons in VD rats.

Our present study had some limitations. The DEPs identified by the proteomic analysis were not verified by immunohistochemistry or Western blotting. In addition, some of the key proteins that mediate the effects of marinoid J during VD require further identification, and their functions should be investigated and clarified in future studies.

## Conclusions

In this study, marinoid J was obtained from the fruits of *Avicennia marina.* Marinoid J effectively ameliorated cognitive deficits in VD rats. Proteomic analysis of rat hippocampal proteins identified 45 DEPs in the marinoid J group compared with those in the VD group. Functional protein-type annotation, functional-type enrichment, and KEGG analysis of these DEPs revealed that marinoid J upregulated the expression of ACE while downregulating the expression of PKCδ proteins, which correlated with amelioration of VD-induced oxidative stress, apoptosis of hippocampal CA1 neurons, and cognitive deficits.

## Supplementary Material

20200806-Supplemental_data.docx
