# Supplementary material for: Marinoid J, a phenylglycoside from Avicennia marina fruit, ameliorates cognitive impairment in rat vascular dementia: a quantitative iTRAQ proteomic study
Source: Pharm Biol. 2020 Dec 5;58(1):1220–9. doi: 10.1080/13880209.2020.1837187 (PMC7723022; doi:10.1080/13880209.2020.1837187)
Supplement: 20200806-Supplemental_data.docx [file IPHB_A_1837187_SM4895.docx]

**Supplemental data**


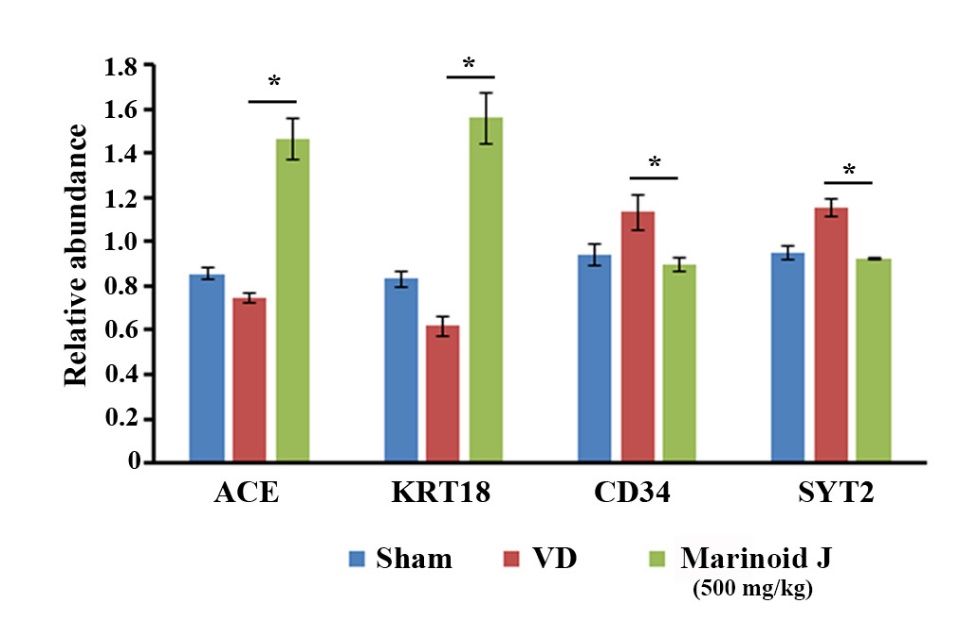


Figure S1. Typical DEPs’ expression pattern in Sham, VD and PGs groups. Values are expressed as means ± SEM (n = 3). *P < 0.05, vs. VD group.


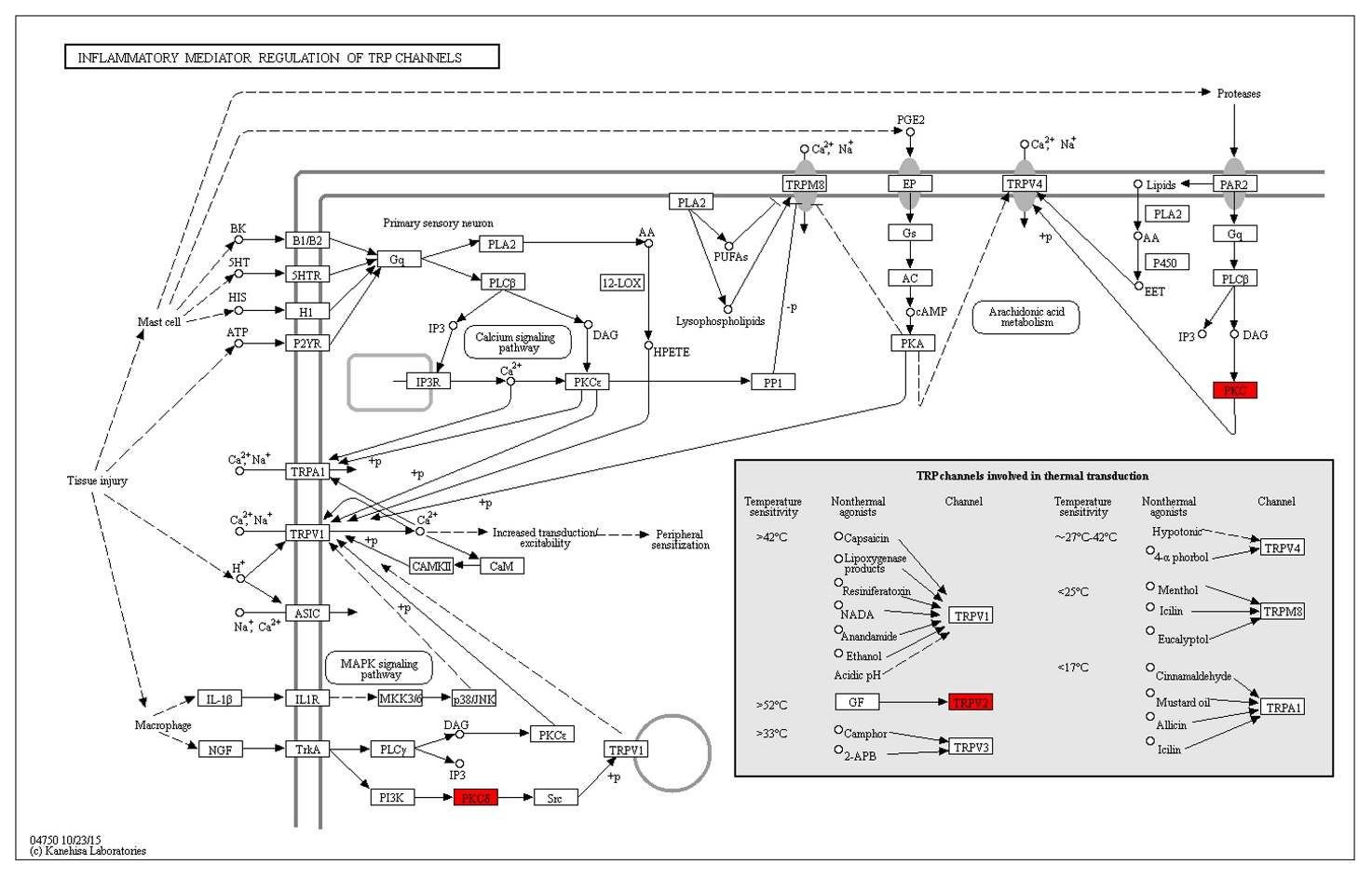


Figure S2. DEPs of the VD group participated in inflammatory mediator regulation of the TRP channels.


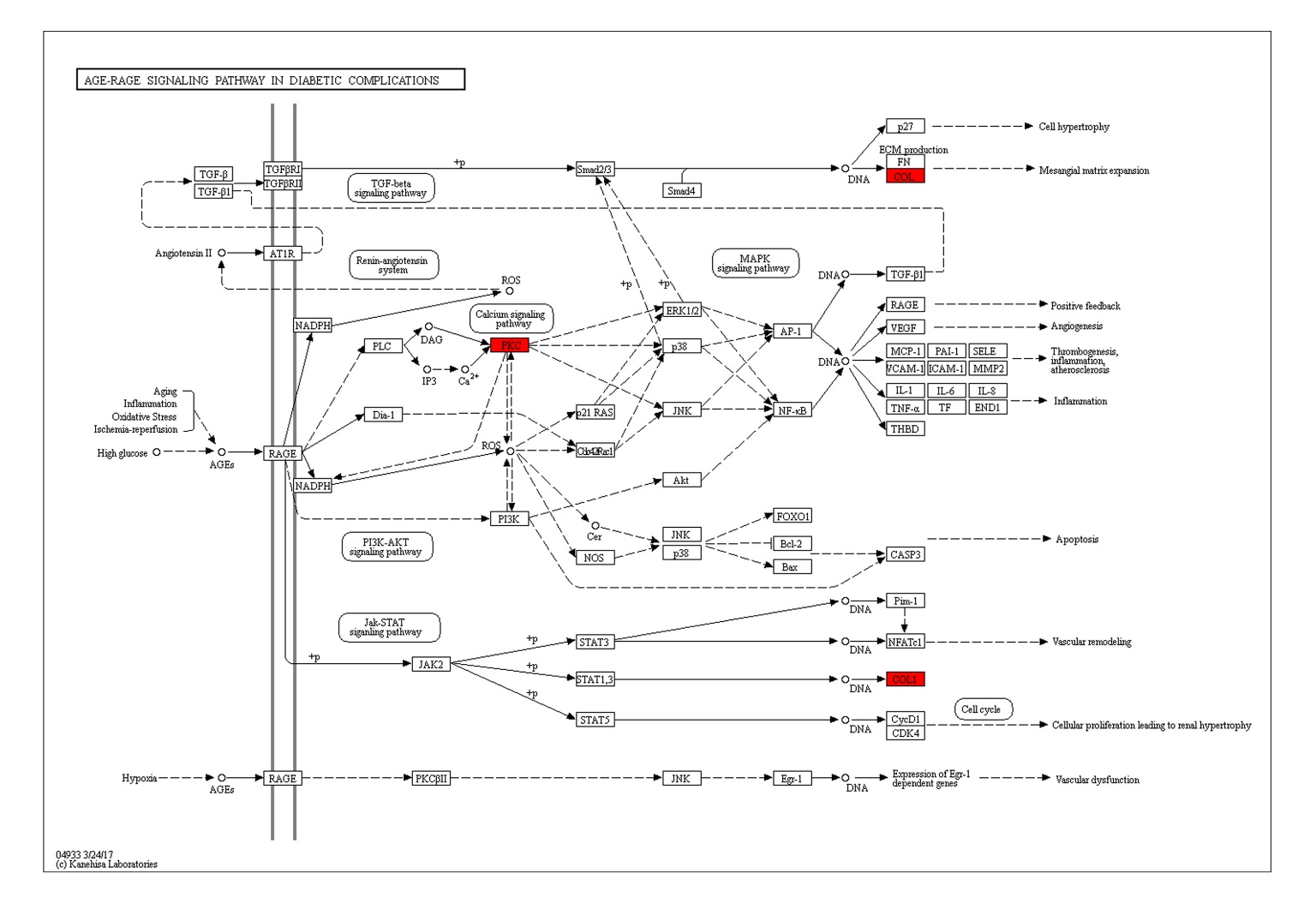


Figure S3. DEPs of the VD group participated in the advanced glycation end product (AGE)-receptor for AGE signaling pathway.


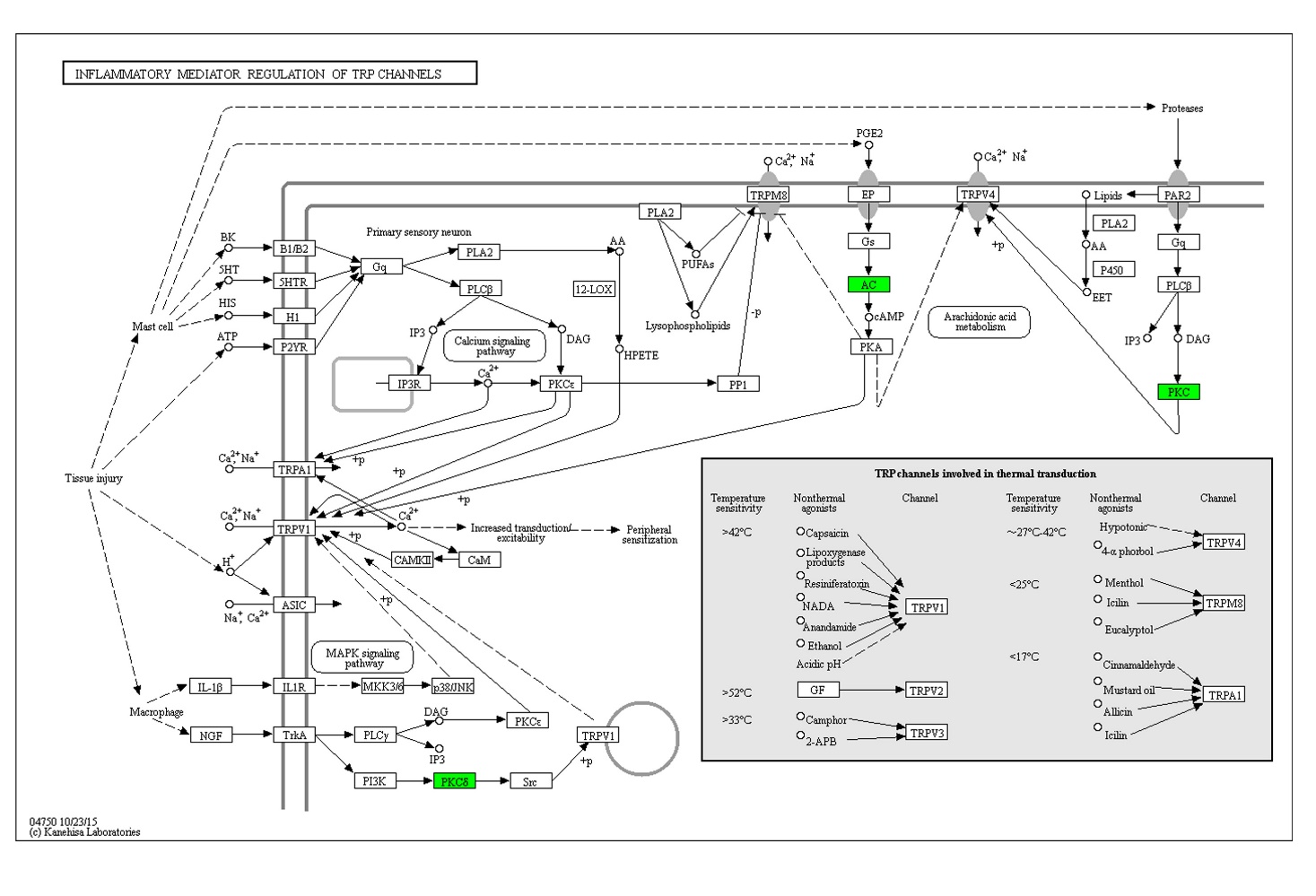


Figure S4. DEPs of the Marinoid J group participated in inflammatory mediator regulation of the TRP channels.


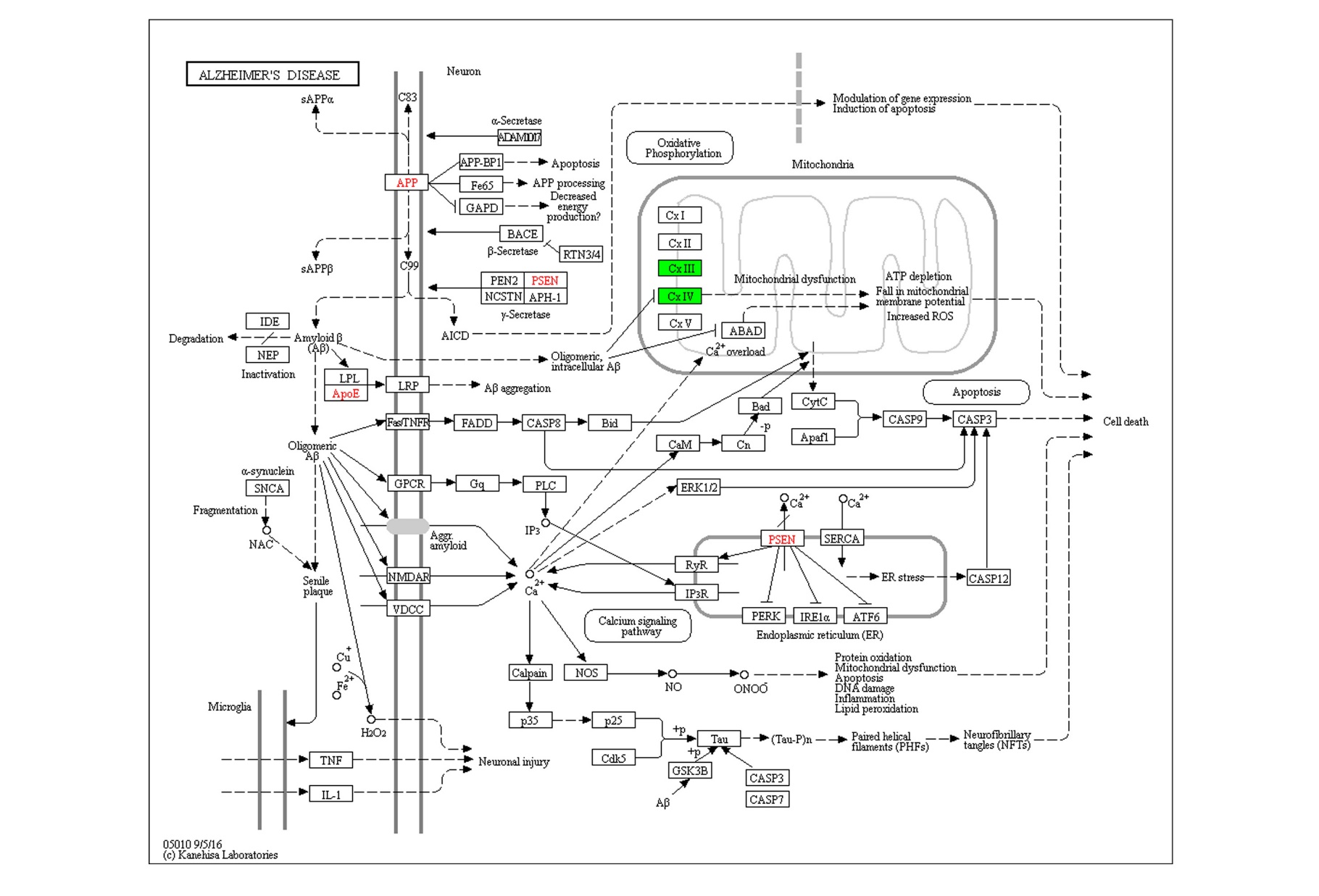


Figure S5. DEPs of the Marinoid J group participated in Alzheimer’s disease.


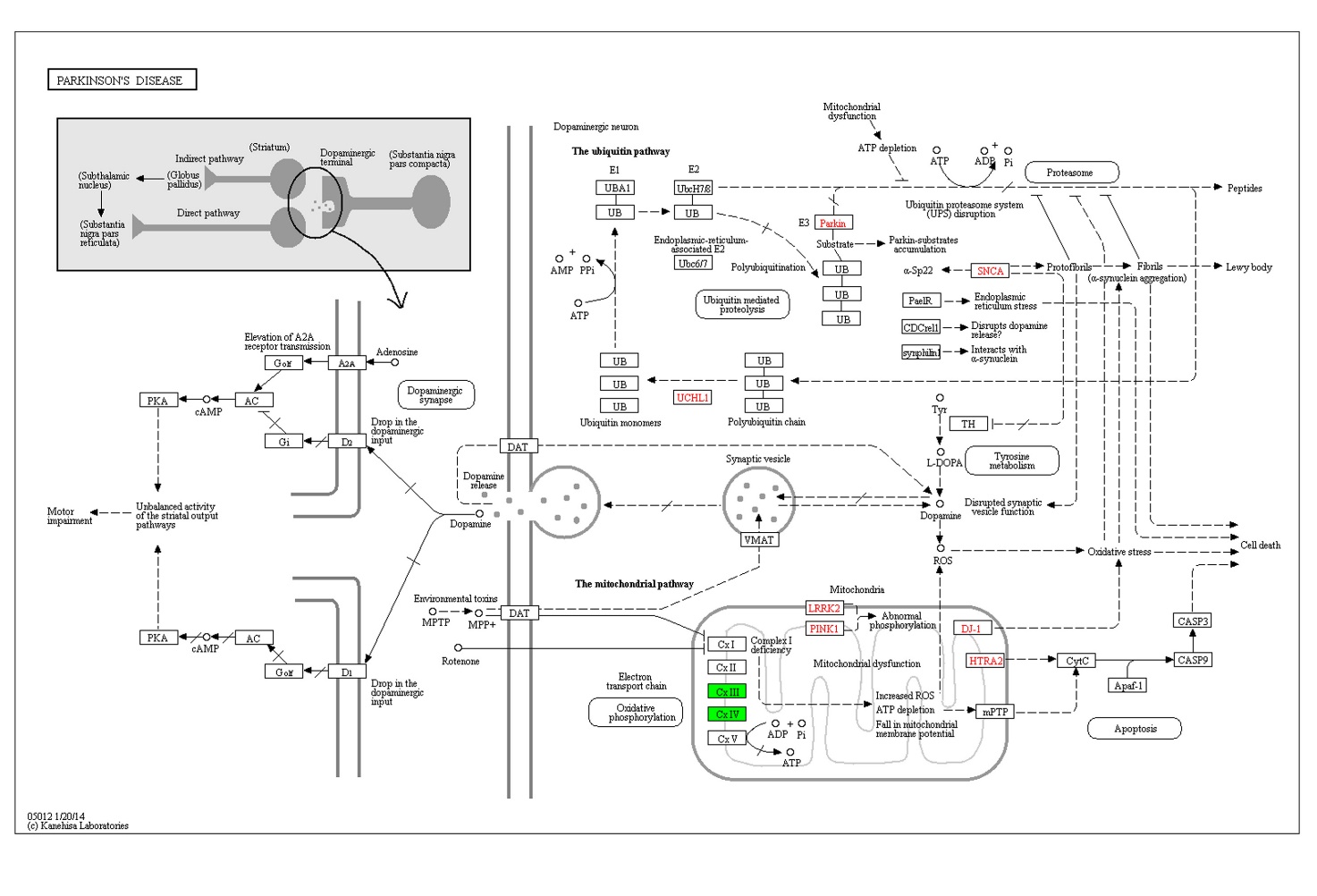


Figure S6. DEPs of the Marinoid J group participated in Parkinson’s disease.


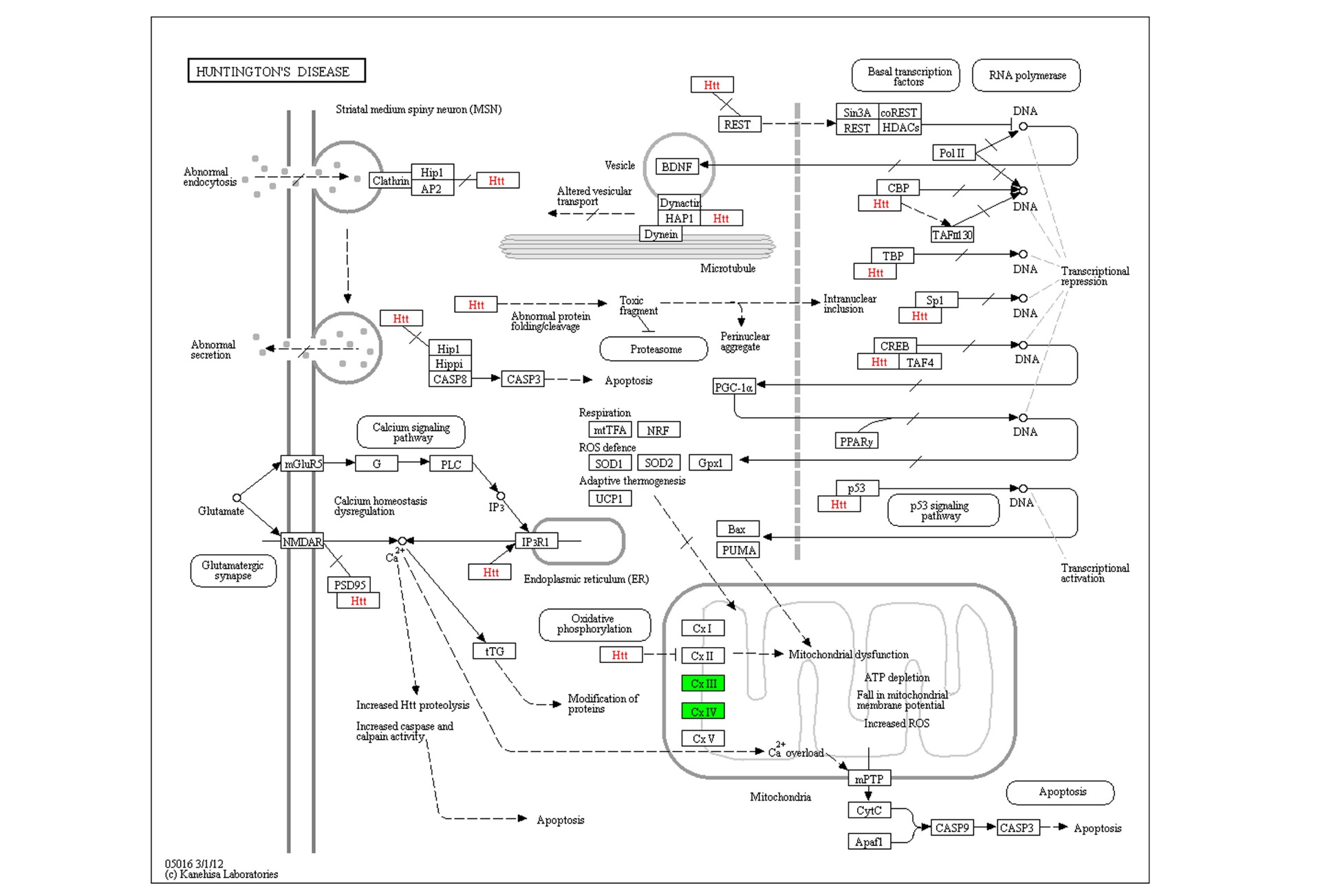


Figure S7. DEPs of the Marinoid J group participated in Huntington’s disease.

In Figure S2 and S3, The red boxes denote upregulated proteins in VD rats.

In Figure S4-7, the full green boxes mean proteins are downregulated in the pathways after Marinoid J treatment, proteins with red color mean they are upregulated in the pathway.
